# Neutrophil-albumin ratio as a biomarker for postoperative complications and long-term prognosis in patients with colorectal cancer undergoing surgical treatment

**DOI:** 10.3389/fnut.2022.976216

**Published:** 2022-11-15

**Authors:** Hailun Xie, Lishuang Wei, Mingxiang Liu, Yanren Liang, Guanghui Yuan, Shunhui Gao, Qiwen Wang, Xin Lin, Shuangyi Tang, Jialiang Gan

**Affiliations:** ^1^Department of Colorectal and Anal Surgery, The First Affiliated Hospital, Guangxi Medical University, Nanning, China; ^2^Guangxi Key Laboratory of Enhanced Recovery After Surgery for Gastrointestinal Cancer, Nanning, China; ^3^Department of Geriatric Respiratory Disease Ward, The First Affiliated Hospital, Guangxi Medical University, Nanning, China; ^4^Grade 2018, Department of Clinical Medicine, Guangxi Medical University, Nanning, China; ^5^Department of Pharmacy, The First Affiliated Hospital, Guangxi Medical University, Nanning, China

**Keywords:** neutrophil-albumin ratio, systemic inflammation, nutrition, colorectal cancer, complication, prognosis

## Abstract

**Background:**

To explore the prognostic value of the preoperative neutrophil-albumin ratio (NAR) in patients with colorectal cancer (CRC) undergoing surgical treatment.

**Materials and methods:**

The standardized log-rank statistic was used to determine the optimal cut-off value for NAR. A logistic regression model was used to evaluate the value of NAR in predicting postoperative complications. Cox proportional hazards models were used to assess the independent association of NAR with progression-free survival (PFS) and overall survival (OS) in CRC patients. Restricted cubic splines were used to assess the relationship between continuous NAR and survival in CRC patients. The Kaplan–Meier method and log-rank test were used to compare survival differences between low and high NAR groups. NAR-based prognostic nomograms were constructed to predict the 1–5-year PFS and OS of CRC patients. The concordance index (C-index) and calibration curve were used to evaluate the prognostic accuracy of the nomograms.

**Results:**

A total of 1,441 CRC patients were enrolled from January 2012 to December 2016. There were 904 men (62.7%) and 537 women (37.3%), with an average age of 58.12 ± 13.15 years. High NAR was closely associated with low BMI, advanced pathological stage, colon cancer, large tumors, vascular invasion, poor differentiation, high CEA levels, long hospital stay, and recurrence and metastasis. A high NAR was an independent risk factor for postoperative complications in CRC patients (OR: 2.298, 95% CI: 1.642–3.216, *p* < 0.001). Patients with a high NAR had worse PFS (40.7 vs. 59.5%, *p* < 0.001) and OS (42.6 vs. 62.4%, *p* < 0.001). After adjusting for confounders, high NAR was independently associated with PFS (HR: 1.280, 95% CI: 1.031–1.589, *p* = 0.025) and OS (HR: 1.280; 95% CI: 1.026–1.596, *p* = 0.029) in CRC patients. The C-index and calibration curves showed that the NAR-based prognostic nomograms had good predictive accuracy.

**Conclusion:**

High NAR was an independent risk factor for postoperative complications and long-term prognosis of CRC patients. NAR-based research could provide references for prognostic judgment and clinical decision-making of CRC patients.

## Introduction

Colorectal cancer (CRC) is a common malignancy of the gastrointestinal tract. CRC had the third highest incidence and second highest mortality among all cancers worldwide, according to the latest data (1). In China, the incidence of CRC ranks fourth and mortality ranks fifth among all malignancies (2). CRC causes a serious social burden, and the prevention and treatment of CRC has become an important public health problem. Therefore, there is an urgent need to identify effective prognostic biomarkers to improve the survival of patients with CRC.

Serological biomarkers have attracted increasing attention because of their simplicity and ease of availability. Serum markers can be used to predict patient prognosis and treatment effects and to formulate individualized treatment interventions that play an important role in the treatment and prognosis evaluation of CRC (3–6). As is known to all, systemic inflammation, as a leading factor in the tumorigenesis process, plays a crucial role and actively participates in the occurrence and development of malignancies (7, 8). In clinical practice, peripheral blood parameters are used as direct indicators of the host environment. Systemic inflammation can be reflected by peripheral blood parameters such as neutrophils, lymphocytes, monocytes, and albumin.

Recently, the newly developed neutrophil-albumin ratio (NAR) has been used to assess prognosis in a variety of diseases, including cerebrovascular disease, pancreatic cancer, and non-small cell lung cancer (9–11). NAR is a novel marker of systemic inflammation and disease severity, which can be calculated using peripheral serum markers (neutrophils and albumin) and has the advantages of being simple, inexpensive, and non-invasive. Neutrophils play an important role in tumorigenesis and tumor progression. A high neutrophil count is thought to be closely associated with poor prognosis of malignancy. Neutrophils can secrete cytokines and chemokines to create a tumor microenvironment suitable for tumor cell proliferation, invasion, and microvascular formation, thereby promoting tumor development and progression (12, 13). Albumin is the most abundant protein in the extracellular matrix synthesized in liver tissue. Decreased albumin level is associated with malnutrition and cancer progression (14–16). Recently, serum albumin was reported to play an important role in systemic inflammation. The decrease in serum albumin may be the result of a combination of protein synthesis recombination in the liver and albumin redistribution in and out of the blood vessels under high systemic inflammation conditions (17). NAR, which combines the advantages of neutrophils and albumin, is a promising biomarker for predicting cancer prognosis.

Currently, there are few studies on the relationship between preoperative NAR and prognosis of patients with CRC. NAR is an emerging indicator of CRC. Therefore, this single-center retrospective study aimed to explore the prognostic value of preoperative NAR in patients with CRC undergoing surgical treatment.

## Patients and methods

### Study population

This cross-sectional retrospective study recruited patients with CRC who underwent surgical treatment at the Colorectal and Anal Surgery Department of The First Affiliated Hospital of Guangxi Medical University from January 2012 to December 2016. Patient information was anonymized during the study period. All enrolled patients met the following inclusion criteria: diagnosis of CRC based on histological or cytological evidence, curative surgery for treatment purposes, and complete preoperative serological data. Patients with multiple primary malignancies, preoperative neoadjuvant chemoradiotherapy, or clinical evidence of infection or other inflammatory diseases were excluded. This study was approved by the ethics review committee of the center. Written informed consent was obtained from all the patients or their close relatives. This study was conducted in strict accordance with the principles of the Declaration of Helsinki.

### Data collection

The following clinicopathological data were collected: sex, age, height, weight, hypertension, diabetes, neutrophil count, albumin level, serum CEA level, T stage, N stage, metastasis, tumor-node-metastasis (TNM) stage, perineural invasion, vascular invasion, pathological type, differentiation, tumor location, tumor size, and surgical approach (laparoscopic or open). Blood characteristics were collected from blood tests performed within 1 week before surgery. All pathological characteristics were obtained from the evaluation of the excised tissue samples by professional pathologists. TNM stage was classified according to the eighth edition of the Union for International Cancer Control (UICC) Pathology classification. The NAR was defined as neutrophil (10^9^)/albumin (g/dL). Body mass index (BMI) was defined as weight (kg)/square height (m^2^) (low, <18.5; normal, 18.5–24; high, ≥24).

### Follow-up and outcomes

In this study, the survival status of all the patients was determined through an outpatient clinic visit or telephone call. Follow-up was performed every 3–6 months in the first year after surgery and every 6–12 months in the second year, until the patient died. The main contents of the follow-up were basic living conditions, serological tests, tumor marker tests, imaging tests, and colonoscopy after surgery. The last follow-up was on July 31, 2021. In this study, the primary outcome was overall survival (OS), and secondary outcomes were progression-free survival (PFS) and postoperative complications. OS was defined as the time interval from the date of diagnosis to death from any cause or the date of the last follow-up. PFS was defined as the time interval from tumor resection to the first recurrence, death, or last follow-up.

### Statistical analysis

Categorical variables were expressed as counts (percentages) and analyzed using Pearson’s chi-squared test or Fisher’s exact test. Continuous variables are expressed as mean (standard deviation) or median (interquartile range) and were analyzed using a *t*-test or non-parametric test. The standardized log-rank statistic was used to determine the optimal cut-off value for NAR by “survminer” R package. Restricted cubic splines (RCS) were used to assess the relationship between NAR and survival of patients with CRC. The Kaplan–Meier method was used to describe survival curves, and the log-rank test was used to compare differences in survival. Univariate and multivariate analyses were performed using the Cox proportional hazards model to evaluate the important factors affecting patient prognosis. Survival risks are expressed as hazard ratios (HRs) and 95% confidence intervals (CIs). The R package “survival” was used to construct prognostic nomograms to predict 1–5-year PFS and OS in patients with CRC. The concordance index (C-index) and calibration curve were used to evaluate the prognostic accuracy of the nomograms. Time-dependent receiver operating characteristic (ROC) curves were used to compare the ability to predict prognosis. A logistic regression model was used to identify the risk factors for complications. Predicted risks are expressed as odds ratios (ORs) and 95% CI. Finally, we randomly divided the total population into two internal validation datasets at a 7:3 ratio to evaluate the generalizability of the results. All p-values were two-sided, and *p*-values below 0.05 were considered statistically significant. R (version 4.0.2) was used for all analyses.

## Results

### Clinicopathologic characteristics

In total, 1,441 patients with CRC were included in this study. There were 904 men (62.7%) and 537 women (37.3%), with an average age of 58.12 ± 13.15 years. A total of 284 patients (19.7%) had stage I, 480 (33.3%) had stage II, 540 (37.5%) had stage III, and 137 (9.5%) had stage IV disease. At the last follow-up, 400 (27.8%) patients had recurrence and metastasis and 582 (40.4%) patients had died. The median follow-up time was 65.23 months (1–106 months). The optimal cut-off value for NAR in patients with CRC was 1.65 (Supplementary Figure 1). Based on this cut-off value, 1,237 patients were identified as having low NAR and 204 patients as having high NAR. We found that high NAR was closely associated with low BMI, metastasis, advanced pathological stage, colon cancer, large tumors, vascular invasion, poor differentiation, and high CEA levels. In addition, patients with CRC with high NAR had a hospital stay that was nearly 3 days longer, a higher risk of recurrence and metastasis, and a higher risk of death (Table 1).

**TABLE 1:** The relationships between the neutrophil-albumin ratio (NAR) and clinicopathological factors of colorectal cancer (CRC) patients.

Features	Overall (*n* = 1,441)	NAR		*P-value*
			
		Low (*n* = 1,237)	High (*n* = 204)	
Gender (male)	904 (62.7)	767 (62.0)	137 (67.2)	0.183
Age (≥60)	724 (50.2)	616 (49.8)	108 (52.9)	0.449
Age [mean (SD)]	58.12 (13.15)	58.15 (13.07)	57.93 (13.67)	0.819
BMI (median [IQR])	22.04 (19.95, 24.31)	22.14 (20.00, 24.46)	21.5(19.32, 23.74)	0.005
**BMI**				0.003
	182 (12.6)	142 (11.5)	40 (19.6)	
	854 (59.3)	736 (59.5)	118 (57.8)	
	405 (28.1)	359 (29.0)	46 (22.5)	
Hypertension (Yes)	241 (16.7)	202 (16.3)	39 (19.1)	0.375
Diabetes (Yes)	90 (6.2)	72 (5.8)	18 (8.8)	0.137
Liver disease (Yes)	56 (3.9)	49 (4.0)	7 (3.4)	0.717
**T stage**				0.232
	50 (3.5)	46 (3.7)	4 (2.0)	
	318 (22.1)	279 (22.6)	39 (19.1)	
	770 (53.4)	660 (53.4)	110 (53.9)	
	303 (21.0)	252 (20.4)	51 (25.0)	
**N stage**				0.158
	808 (56.1)	705 (57.0)	103 (50.5)	
	398 (27.6)	338 (27.3)	60 (29.4)	
	235 (16.3)	194 (15.7)	41 (20.1)	
Clinical distant metastasis (Yes)	137 (9.5)	97 (7.8)	40 (19.6)	<0.001
TNM stage				<0.001
	284 (19.7)	254 (20.5)	30 (14.7)	
	480 (33.3)	420 (34.0)	60 (29.4)	
	540 (37.5)	466 (37.7)	74 (36.3)	
	137 (9.5)	97 (7.8)	40 (19.6)	
Perineural invasion (Yes)	149 (10.3)	128 (10.3)	21 (10.3)	0.999
Vascular invasion (Yes)	247 (17.1)	201 (16.2)	46 (22.5)	0.035
**Macroscopic type**				0.001
Protrude type	406 (28.2)	335 (27.1)	71 (34.8)	
Infiltrating type	113 (7.8)	88 (7.1)	25 (12.3)	
Ulcerative type	922 (64.0)	814 (65.8)	108 (52.9)	
Differentiation (Poor)	190 (13.2)	147 (11.9)	43 (21.1)	<0.001
Tumor location (Rectal)	736 (51.1)	670 (54.2)	66 (32.4)	<0.001
Tumor size (median [IQR])	4.50 (3.50, 6.00)	4.20 (3.50, 5.50)	6.00 (4.00, 8.00)	<0.001
CEA (High)	594 (41.2)	483 (39.0)	111 (54.4)	<0.001
Surgical method (Endoscopic)	837 (58.1)	774 (62.6)	63 (30.9)	<0.001
Operation time (median [IQR])	188.00 (150.00, 245.00)	183.00 (146.00, 240.00)	216.50(172.75, 278.25)	<0.001
Intraoperatve blood loss (median [IQR])	100.00 (50.00, 200.00)	100.00 (50.00, 200.00)	200.00 (100.00, 300.00)	<0.001
Length of stay (median [IQR])	17.00 (11.00, 21.00)	16.00 (11.00, 20.00)	19.00 (14.00, 24.00)	<0.001
Radiotherapy (Yes)	134 (9.3)	125 (10.1)	9 (4.4)	0.014
Chemotherapy (Yes)	657 (45.6)	571 (46.2)	86 (42.2)	0.323
Recurrence and metastasis (Yes)	400 (27.8)	330 (26.7)	70 (34.3)	0.024
Death (Yes)	582 (40.4)	465 (37.6)	117 (57.4)	<0.001

CRC, colorectal cancer; BMI, body mass index; NAR, neutrophil-albumin ratio.

### The relationship between neutrophil-albumin ratio and complications

A total of 299 patients (26.2%) developed varying degrees of postoperative complications. According to the modified Clavien complication classification system, there were 147 (10.2%) grade I complications, 108 (7.5%) grade II complications, 17 (1.2%) grade IIIa complications, 13 (0.9%) grade IIIb complications, seven (0.5%) grade IVa complications, six (0.4%) grade IVb complications, and one (0.1%) grade V complication. Compared with the low-NAR group, CRC patients in the high-NAR group had a significantly higher incidence of total postoperative complications, especially grade I–III complications (Supplementary Table 1). Logistic regression analysis showed that high NAR was an independent risk factor for postoperative complications in patients with CRC. Compared with patients with low NAR, patients with high NAR had a 1.298-fold higher risk of postoperative complications (OR: 2.298, 95% CI: 1.642–3.216, *p* < 0.001) (Supplementary Table 2).

### Comparison of the survival differences between the low and high neutrophil-albumin ratio groups

**FIGURE 1:**
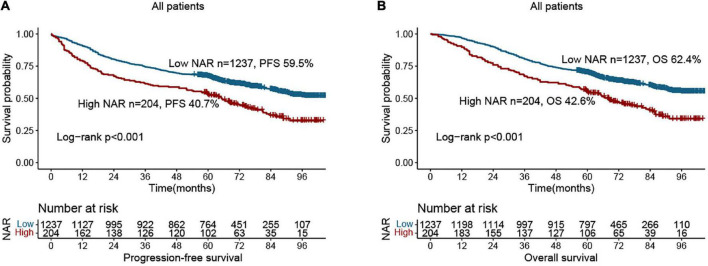
Kaplan-Meier curve of neutrophil-albumin ratio (NAR) in colorectal cancer (CRC) patients. **(A)** Progression-free survival; **(B)** overall survival.

**FIGURE 2:**
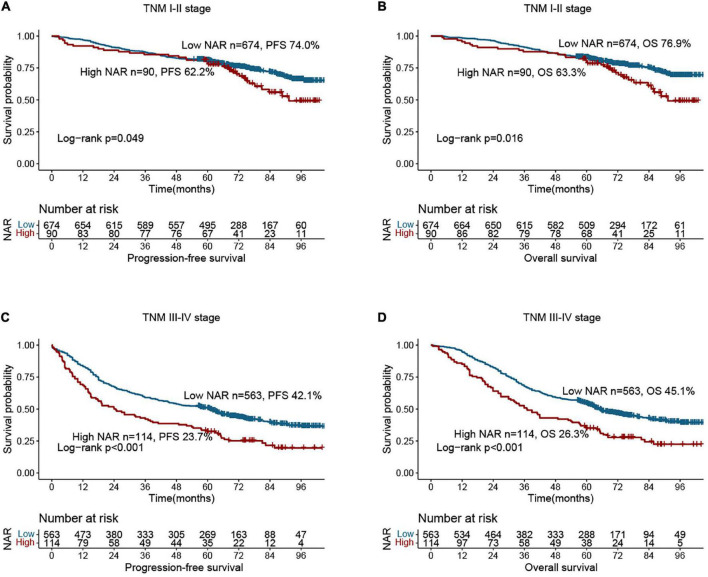
Stratified survival analysis of neutrophil-albumin ratio (NAR) based on tumor-node-metastasis (TNM) stage. **(A)** Progression-free survival of TNM I-II stage; **(B)** overall survival of TNM I-II stage; **(C)** overall survival of TNM III-IV stage; **(D)** overall survival of TNM III-IV stage.

Compared with patients with low NAR, those with high NAR had worse PFS (40.7 vs. 59.5%, *p* < 0.001) (Figure 1A). Patients with a high NAR had significantly worse OS than those with a low NAR (42.6 vs. 62.4%, *p* < 0.001) (Figure 1B). In addition, we also performed subgroup analysis of different CEA levels, and found that regardless of whether CEA levels were normal or high, the PFS and OS of patients in the high NAR group were significantly lower than those of patients in the low NAR group (Supplementary Figure 2). Notably, NAR can effectively stratify the prognosis of patients with CRC at different pathological stages. For early stages (TNM stage I–II), the PFS and OS of the high NAR group were significantly poorer than those of the low NAR group (Figures 2A,B). For advanced stages (TNM stage III–IV), NAR still provides effective prognostic differentiation and has stronger discriminative power in patients with CRC (Figures 2C,D).

### Relationship between neutrophil-albumin ratio and survival

**FIGURE 3:**
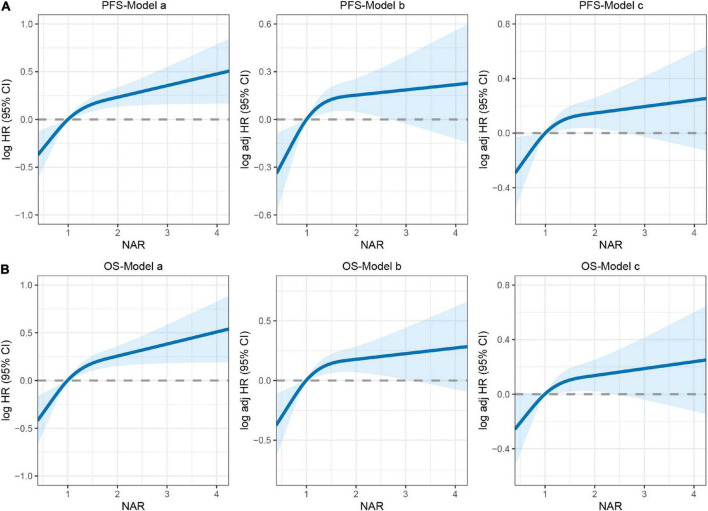
The association between neutrophil-albumin ratio (NAR) and survival in patients with colorectal cancer (CRC). **(A)** Progression-free survival; **(B)** overall survival. Model a: no adjusted. Model b: adjusted for gender, age, and BMI. Model c: adjusted for gender, age, BMI, hypertension, diabetes, T stage, N stage, metastasis, tumor location, tumor size, perineural invasion, vascular invasion, macroscopic type, differentiation, surgical approach, operating time, blood loss.

There was a clear dose-response relationship between NAR and survival in patients with CRC under different adjustment models, and NAR was inversely associated with prognosis (Figures 3A,B). After adjusting for confounders, high NAR was independently associated with PFS (HR: 1.280, 95% CI: 1.031–1.589, *p* = 0.025) and OS (HR: 1.280, 95% CI: 1.026–1.596, *p* = 0.029) in patients with CRC (Tables 2, 3). We also conducted a trend test for the relationship between NAR and PFS/OS. The results showed that NAR was independently associated with PFS/OS and OS in patients with CRC, either as a continuous variable or as a categorical variable (Supplementary Table 3). In addition, high NAR was a risk factor affecting the vast majority of patient subgroups (Supplementary Figure 3).

**TABLE 2:** Univariate and multivariate Cox regression analysis of clinicopathological characteristics associated with progression-free survival (PFS) in colorectal cancer (CRC) patients.

Characteristic	Progression-free survival			
	
	Univariate analysis		Multivariate analysis	
		
	HR (95% CI)	*P-value*	HR (95% CI)	*P-value*
Gender (Female)	1.014 (0.862–1.193)	0.866		
Age (≥60 years)	1.28 (1.093–1.499)	0.002	1.32 (1.122–1.554)	0.001
BMI		0.108		0.783
Low	Ref.		Ref.	
Normal	0.871 (0.694–1.095)	0.237	0.938 (0.741–1.187)	0.595
High	0.762 (0.589–0.986)	0.038	0.91 (0.697–1.188)	0.486
Hypertension (Yes)	1.198 (0.979–1.466)	0.079		
Diabetes (Yes)	1.152 (0.845–1.57)	0.371		
NAR (High)	1.703 (1.396–2.077)	<0.001	1.280 (1.031–1.589)	0.025
T stage (T3–4)	2.364 (1.896–2.947)	<0.001	1.426 (1.123–1.811)	0.004
N stage		<0.001		<0.001
N0	Ref.		Ref.	
N1	1.872 (1.553–2.257)	<0.001	1.531 (1.261–1.859)	<0.001
N2	4.055 (3.338–4.927)	<0.001	2.798 (2.254–3.472)	<0.001
Distant metastasis (Yes)	5.384 (4.411–6.572)	<0.001	3.048 (2.442–3.805)	<0.001
Tumor location (Colon)	0.918 (0.784–1.075)	0.287		
Tumor size (≥5 cm)	1.192 (1.019–1.395)	0.028	0.96 (0.814–1.132)	0.627
Perineural invasion (Positive)	1.755 (1.403–2.195)	<0.001	1.127 (0.877–1.447)	0.352
Vascular invasion (Positive)	1.997 (1.663–2.397)	<0.001	1.21 (0.978–1.496)	0.080
Macroscopic type		0.003		0.313
Protrude type	Ref.			
Infiltrating type	1.466 (1.075–1.998)	0.016	1.214 (0.887–1.662)	0.225
Ulcerative type	1.366 (1.129–1.653)	0.001	1.151 (0.944–1.403)	0.165
Differentiation (High/Medium)	0.7 (0.563–0.869)	0.001	0.869 (0.693–1.091)	0.228
Surgical approach (Laparoscope)	0.673 (0.575–0.788)	<0.001	0.882 (0.741–1.051)	0.161
Operating time (median) (≥192 min)	1.205 (1.029–1.412)	0.021	1.048 (0.887–1.239)	0.581
Blood loss (median) (≥100 ml)	1.246 (1.052–1.477)	0.011	1.078 (0.901–1.291)	0.412
CEA (≥5 ng/ml)	1.988 (1.698–2.328)	<0.001	1.474 (1.245–1.746)	<0.001
Radiotherapy (Yes)	1.151 (0.885–1.496)	0.293		
Chemotherapy (Yes)	1.141 (0.974–1.336)	0.101		

CRC, colorectal cancer; BMI, body mass index; NAR, neutrophil-albumin ratio.

**TABLE 3:** Univariate and multivariate Cox regression analysis of clinicopathological characteristics associated with overall survival (OS) in colorectal cancer (CRC) patients.

Characteristic	Overall survival			
	
	Univariate analysis		Multivariate analysis	
		
	HR (95% CI)	*P-value*	HR (95% CI)	*P-value*
Gender (Female)	1.013 (0.857–1.198)	0.881		
Age (≥60 years)	1.344 (1.141–1.583)	<0.001	1.356 (1.145–1.606)	<0.001
BMI		0.060		0.579
Low	Ref.		Ref.	
Normal	0.858 (0.678–1.085)	0.200	0.919 (0.721–1.171)	0.492
High	0.73 (0.56–0.952)	0.020	0.864 (0.656–1.138)	0.299
Hypertension (Yes)	1.202 (0.976–1.48)	0.084		
Diabetes (Yes)	1.188 (0.865–1.632)	0.287		
NAR (High)	1.769 (1.444–2.167)	<0.001	1.280 (1.026–1.596)	0.029
pT stage (T3–4)	2.495 (1.975–3.151)	<0.001	1.464 (1.138–1.885)	0.003
pN stage		<0.001		<0.001
N0	Ref.		Ref.	
N1	1.875 (1.544–2.277)	<0.001	1.519 (1.242–1.859)	<0.001
N2	4.079 (3.339–4.983)	<0.001	2.658 (2.127–3.32)	<0.001
Distant metastasis (Yes)	5.609 (4.581–6.866)	<0.001	3.186 (2.546–3.987)	<0.001
Tumor location (Colon)	0.976 (0.83–1.149)	0.772		
Tumor size (≥5 cm)	1.308 (1.112–1.539)	0.001	1.069 (0.901–1.267)	0.444
Perineural invasion (Positive)	1.713 (1.359–2.159)	<0.001	1.075 (0.829–1.393)	0.587
Vascular invasion (Positive)	2.039 (1.691–2.459)	<0.001	1.256 (1.01–1.562)	0.041
Macroscopic type		0.006		0.399
Protrude type	Ref.			
Infiltrating type	1.448 (1.05–1.997)	0.024	1.175 (0.848–1.627)	0.333
Ulcerative type	1.366 (1.12–1.665)	0.002	1.147 (0.934–1.409)	0.192
Differentiation (High/Medium)	0.648 (0.521–0.807)	0.001	0.789 (0.627–0.993)	0.044
Surgical approach (Laparoscope)	0.648 (0.551–0.763)	<0.001	0.89 (0.743–1.066)	0.207
Operating time (median) (≥192 min)	1.21 (1.028–1.426)	0.022	1.075 (0.904–1.278)	0.413
Blood loss (median) (≥100 ml)	1.263 (1.059–1.506)	0.009	1.104 (0.916–1.331)	0.298
CEA (≥5 ng/ml)	2.036 (1.73–2.397)	<0.001	1.472 (1.236–1.754)	<0.001
Radiotherapy (Yes)	0.939 (0.705–1.252)	0.67		
Chemotherapy (Yes)	1.047 (0.889–1.233)	0.581		

CRC, colorectal cancer; BMI, body mass index; NAR, neutrophil-albumin ratio.

### Construction of prognostic nomograms

**FIGURE 4:**
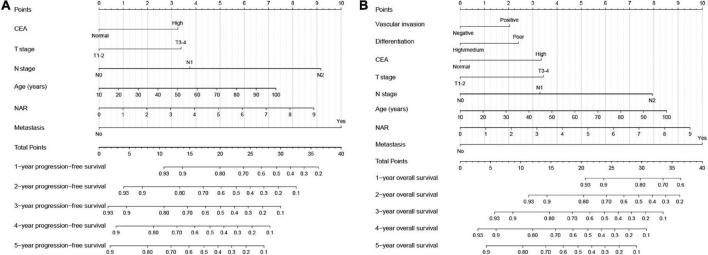
Construction the neutrophil-albumin ratio (NAR)-based prognostic nomograms in colorectal cancer (CRC) patients. **(A)** The progression-free survival nomogram; **(B)** the overall survival nomogram.

Based on the results of the Cox proportional hazards model of PFS, we developed a PFS nomogram to predict postoperative 1–5-year PFS in patients with CRC (Figure 4A), which included age, NAR, T stage, N stage, metastasis, and CEA. Age and NAR were used as continuous variables to improve the predictive accuracy of the nomograms. The nomogram showed that with increasing age/NAR, progression of T stage/N stage, metastasis, and increasing CEA levels, the predicted score increased, indicating that the risk of poor prognosis also increased. In the survival analysis of OS, six factors including age, NAR, T stage, N stage, metastasis, vascular invasion, differentiation, and CEA were confirmed to be independently associated with the prognosis of CRC patients. Therefore, we included these prognostic factors to construct an OS nomogram for predicting postoperative 1–5-year OS in CRC patients (Figure 4B). The nomogram showed that with increasing age/NAR, progression of T stage/N stage, metastasis, poor differentiation, emergence of vascular invasion, and increased CEA, the predicted score increased, indicating that the risk of poor prognosis also increased.

### Utility evaluation of survival nomograms

The C-indices of the PFS and OS nomograms were 0.720 (95% CI: 0.699–0.741) and 0.728 (95% CI: 0.706–0.750), respectively. The calibration curves for both the 3- and 5-year PFS (Supplementary Figures 4A,B) and OS (Supplementary Figures 4C,D) demonstrated the best agreement between the predicted survival probabilities and actual observations. These results demonstrated that the nomograms had good predictive accuracy in predicting the prognosis of patients with CRC. Furthermore, we compared these nomograms with the traditional TNM staging system by using time-dependent ROC curves. The results showed that our nomograms had better resolution and accuracy in predicting 3- and 5-year PFS (Supplementary Figures 5A,B) and OS (Supplementary Figures 5C,D) than TNM stage did.

### Internal validation

**FIGURE 5:**
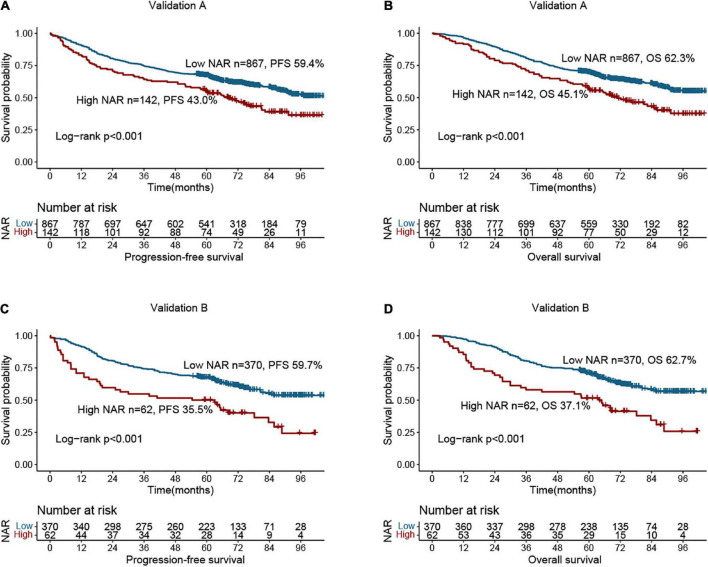
Survival analysis of neutrophil-albumin ratio (NAR) in colorectal cancer (CRC) patients at internal validation cohorts. **(A)** Progression-free survival of NAR at validation A; **(B)** overall survival of NAR at validation A; **(C)** progression-free survival of NAR at validation B; **(D)** overall survival of NAR at validation B.

We performed randomized internal validation by dividing the total population into validation a (1,009) and validation b (432) cohorts at a 7:3 ratio. Supplementary Table 4 compares the clinicopathological factors of the two cohorts, and the results show that the two internal cohorts were independent. NAR still provided a valid prognostic assessment in patients in the validation a (Figures 5A,B) and validation b cohorts (Figures 5C,D). Compared with patients with low NAR, those with high NAR have a higher risk of poor prognosis. Next, we internally validated the PFS and OS nomograms. In validation a, the C-indices of the PFS and OS nomograms were 0.712 (0.688, 0.737) and 0.726 (0.701, 0.751), respectively. In validation b, the C-indices of the PFS and OS nomograms were 0.742 (0.706, 0.778) and 0.738 (0.701, 0.775), respectively. The calibration curves for 3- and 5-year PFS and OS both demonstrated the best agreement between predicted survival probabilities and actual observation in validation a (Supplementary Figure 6A) and validation b (Supplementary Figure 6B).

## Discussion

Systemic inflammation is considered to be the seventh hallmark of cancer and is involved in tumor development, proliferation, metastasis, aging, and apoptosis. Ostan et al. suggested that inflammation triggers genetic mutations or changes in epigenetic mechanisms that promote cancer initiation, metastasis, and progression (18, 19). Changes in inflammatory cells and inflammatory proteins in the peripheral venous blood can reflect tumor progression. Therefore, as markers of systemic inflammation, peripheral venous blood counts and albumin levels may provide additional information about the outcomes of patients with malignancies.

In this study, we found that an elevated preoperative NAR may reflect more aggressive tumor features. Patients with a high preoperative NAR had longer hospital stays and a higher risk of poor prognosis, suggesting that NAR can be used to assess disease burden. In addition, high preoperative NAR was closely associated with postoperative complications, especially grade I–III complications. In our study, approximately 20.7% of patients with CRC had varying degrees of postoperative complications, and the proportion of patients with postoperative complications in the high NAR group was 37.7%, while it was only 17.9% in the low NAR group. Multivariate logistic regression analysis showed that a high preoperative NAR was an independent risk factor for postoperative complications in patients with CRC.

Multivariate RCS showed that with increasing NAR, the prognosis of patients became progressively worse. We found that patients with high NAR had a significantly worse prognosis than those with low NAR. Multivariate survival analysis showed that a high NAR was an independent risk factor for shorter PFS and OS in patients with CRC. The TNM staging system is currently recognized as the most reliable tool for evaluating the prognosis of CRC patients. However, it has been reported that patients with the same TNM stage may still have different clinical outcomes, suggesting that other prognostic indices need to be assessed to achieve a more accurate prognostic evaluation in the setting of the same TNM stage (20). We found that NAR was also effective in the prognostic stratification of different pathological stages, suggesting that it could be an effective complement for evaluating the prognosis of CRC patients with the same pathological stage.

For convenience and intuitive use in clinical studies, we constructed novel and effective prognostic nomograms. These nomograms consist of specific clinical features, each feature corresponding to a specific point, and a score for that feature can be calculated by drawing a straight line on the point axis, and then positioning the sum of these feature scores on the total point axis. The risk probability can then be calculated by plotting down to the prediction axis. These nomograms have the advantage of integrating individual profiles, tumor characteristics, serum tumor markers, and nutritional inflammation-related markers and can be used for personalized assessment of 1–5-year PFS and OS in patients with CRC. The results of the C-index and calibration plots of the overall cohort and internal validation cohorts confirmed the good predictive accuracy of our constructed prognostic nomogram. For patients with higher scores, indicating greater tumor aggressiveness and higher tumor-associated inflammation, closer follow-up monitoring and even more aggressive anticancer therapy could be considered to improve prognosis.

It is well-known that neutrophils are an important defense line of the body’s immunity, and albumin is a commonly used indicator to reflect the nutritional status of patients in clinical practice (21, 22). NAR, combined the parameters of immune and nutrition, comprehensively reflects the perioperative nutritional reserve and anti-attack ability of CRC patients, which may be the reason why low NAR is closely related to postoperative complications of CRC patients. Neutrophils release chemokines and cytokines that play important roles in stimulating angiogenesis, cytogenesis, antiviral defense, and modulating immune responses (12). Serum albumin is associated with activated systemic inflammation during tumor proliferation and invasion (17). Therefore, NAR is not only an index reflecting the nutritional status, but also a novel marker indicating systemic inflammation and disease severity. Systemic inflammation is the basis of cancer development and progression, so NAR has a good predictive effect on the prognosis of CRC patients.

Uludag et al. found that NAR may be a useful predictive marker for advanced colon cancer, providing more detailed prognostic information for patients with colon cancer and physicians (23). Tawfik et al. found that NAR can be a predictor of pathological complete response after neoadjuvant chemoradiotherapy in patients with rectal cancer (24). However, their small sample size and short follow-up period led to certain limitations. In addition, no studies have reported the relationship between preoperative NAR, postoperative complications, and long-term outcomes in patients with CRC. Therefore, this study is the first to report that a high preoperative NAR is an independent risk factor for postoperative complications and prognosis in patients with CRC. Furthermore, we constructed the NAR-based prognostic nomograms that can directly help clinicians quantify the prognostic risk of CRC patients, thereby making it more convenient and personalized to formulate appropriate treatment strategies for CRC patients. However, our study has some limitations. As this was a single-center retrospective study, there may be a potential selection bias, such as selection bias, follow-up bias, etc. Although we performed an internal validation of the nomograms, further validation in external cohorts is required before the nomogram can be used clinically.

## Conclusion

High NAR was an independent risk factor affecting postoperative complications and long-term prognosis of patients with CRC. NAR-based nomograms have good predictive accuracy and can provide a personalized reference for prognostic judgment and clinical decision-making of patients with CRC.

## Data availability statement

The raw data supporting the conclusions of this article will be made available by the authors, without undue reservation.

## Ethics statement

The studies involving human participants were reviewed and approved by the Ethics Committee of The First Affiliated Hospital of Guangxi Medical University, with the approval number: 2021 (KY-E-043). The patients/participants provided their written informed consent to participate in this study.

## Author contributions

JG had full access to all the data in the study, took responsibility for the integrity of the data and the accuracy of the data analysis, and performed conception and design. JG and ST provided management support. HX, GY, and ML did data collection. HX did data analysis and professional drafting. HX and LW wrote the manuscript. All authors agreed to publish, contributed to the manuscript, and approved the submitted version.
