# Supplementary material for: Neutrophil-albumin ratio as a biomarker for postoperative complications and long-term prognosis in patients with colorectal cancer undergoing surgical treatment
Source: Front Nutr. 2022 Nov 15;9:976216. doi: 10.3389/fnut.2022.976216 (PMC9705583; doi:10.3389/fnut.2022.976216)
Supplement: Supplementary file 1 [file Data_Sheet_1.docx]

**Figure S1.** The cur-point of NAR in CRC patients.

**
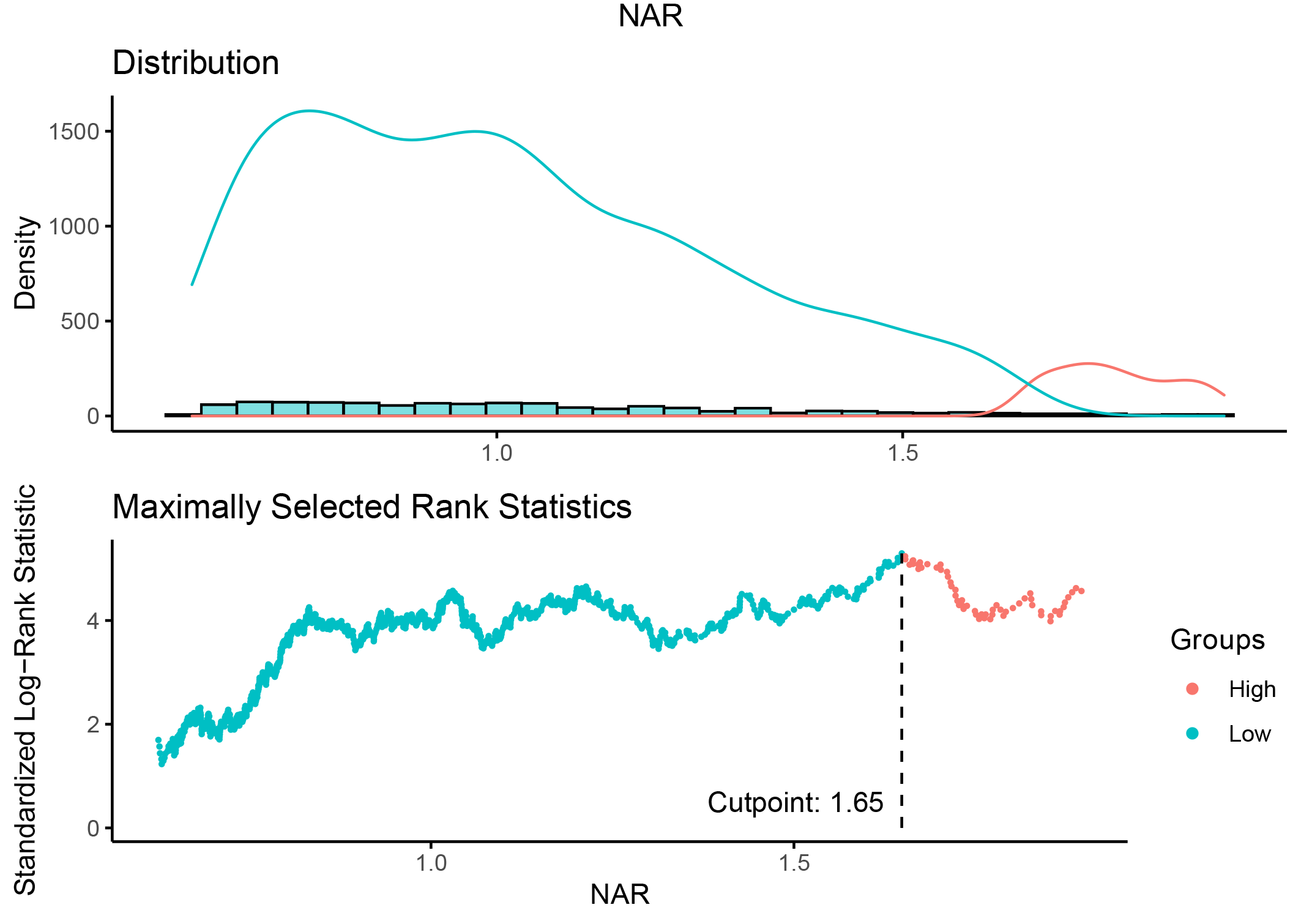
**

**Figure S2.** Stratified survival analysis of NAR based on CEA level.


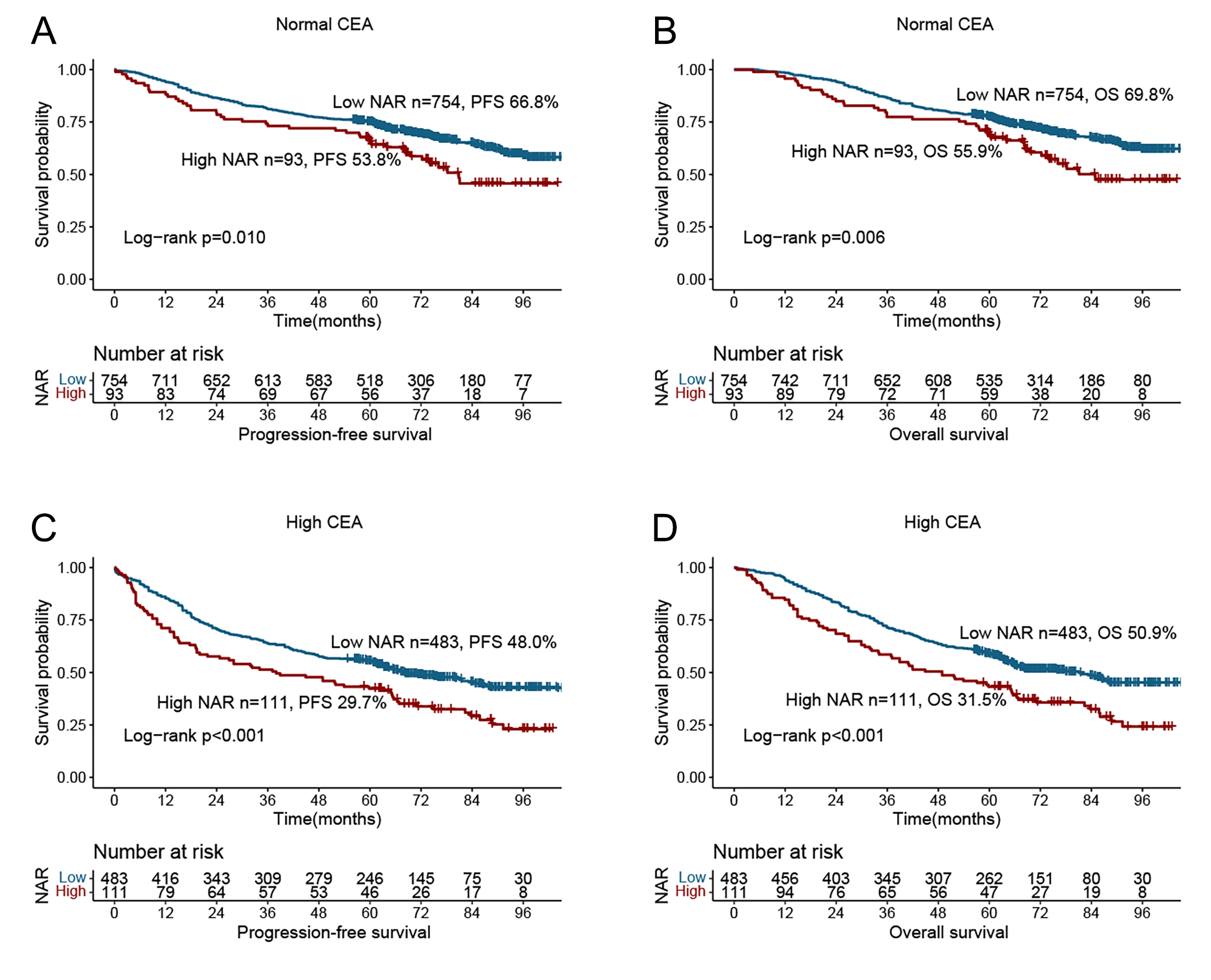


**Notes:** A,Progression-free survival of NAR at normal CEA level; B, Overall survival of NAR at normal CEA level; C, Progression-free survival of NAR at high CEA level; D, Overall survival of NAR at high CEA level.

**Figure S3.** The association between NAR and hazard risk of OS and PFS in various subgroups. (A, Progression-free survival, B, Overall survival).


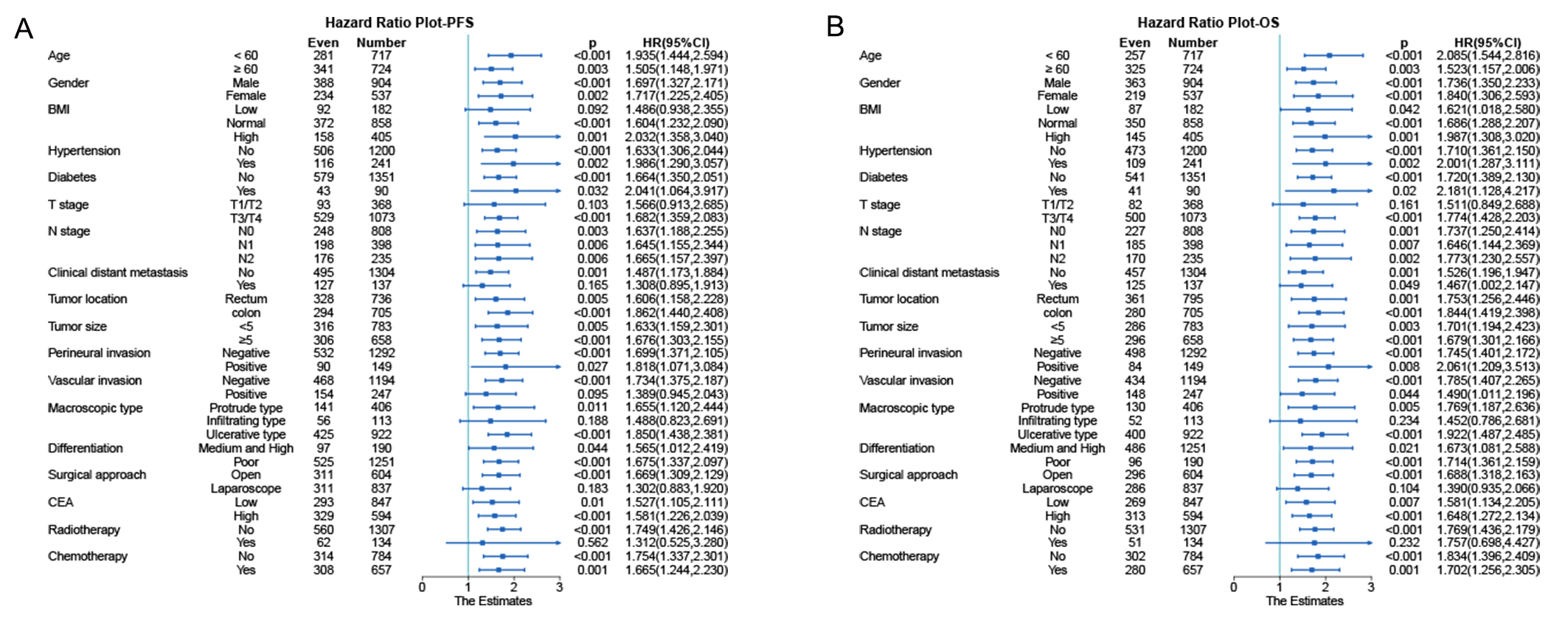


**Figure S4.** Calibration curve of the PFS and OS nomograms.

**
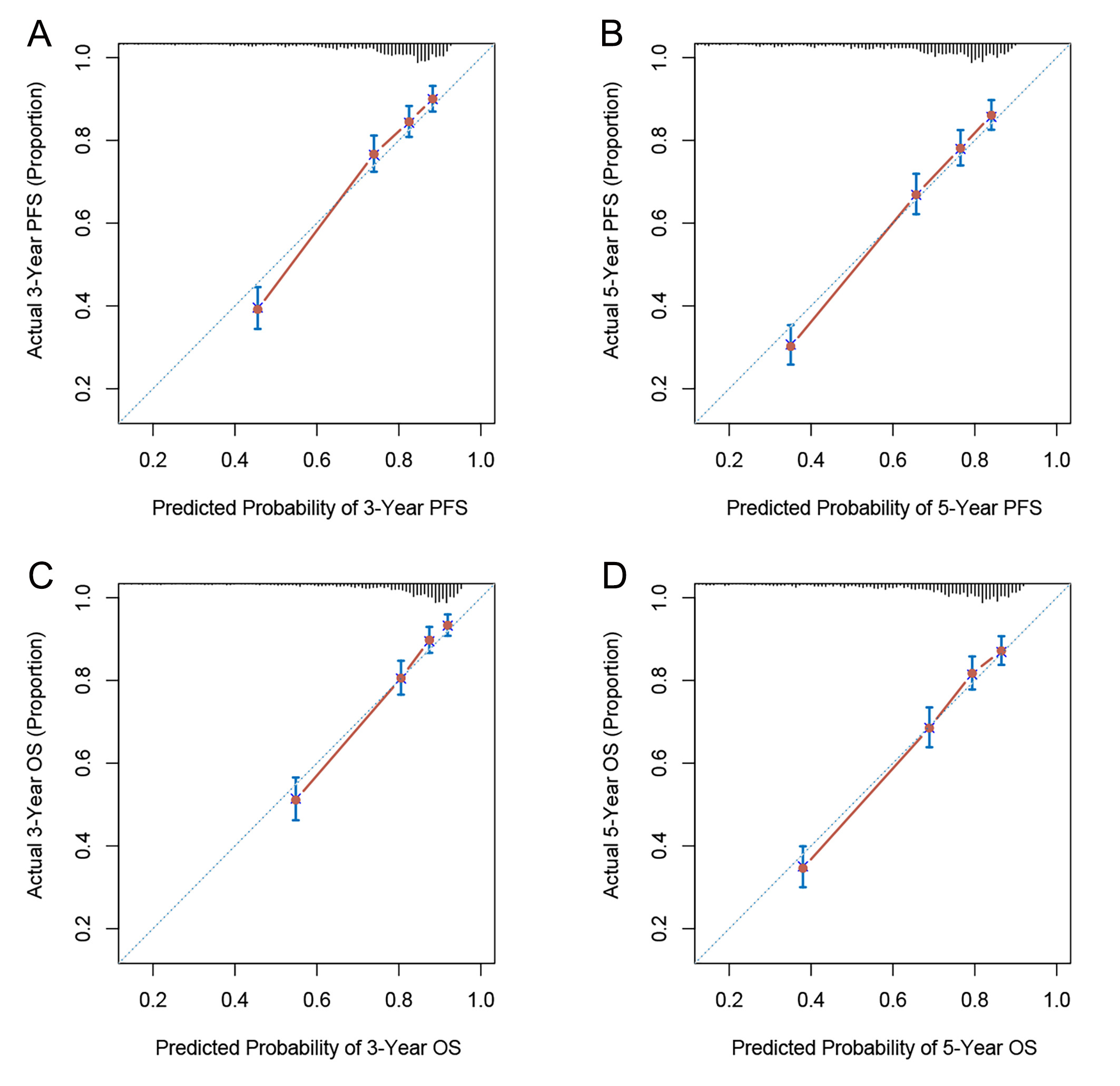
**

**Notes:** A, 3-year PFS; B, 5-year PFS; C, 3-year OS; D, 5-year OS.

**Figure S5.** Comparison of the ability of the novel prognostic nomograms and TNM classification in predicting PFS and OS of CRC patients.

**
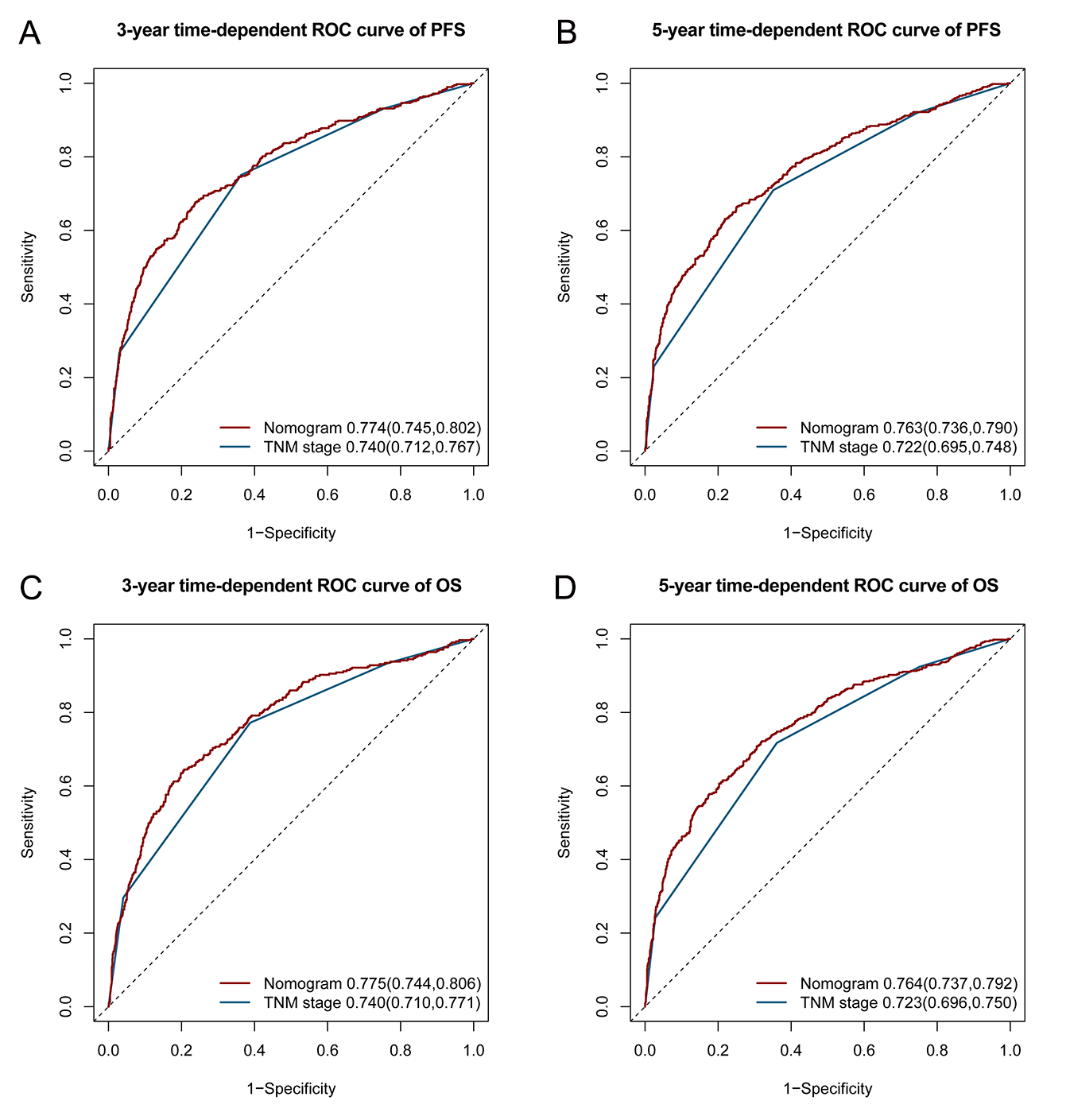
**

**Notes:** A, 3-year PFS; B, 5-year PFS; C, 3-year OS; D, 5-year OS.

**Figure S6.** Calibration curve at randomize internal validations.

**
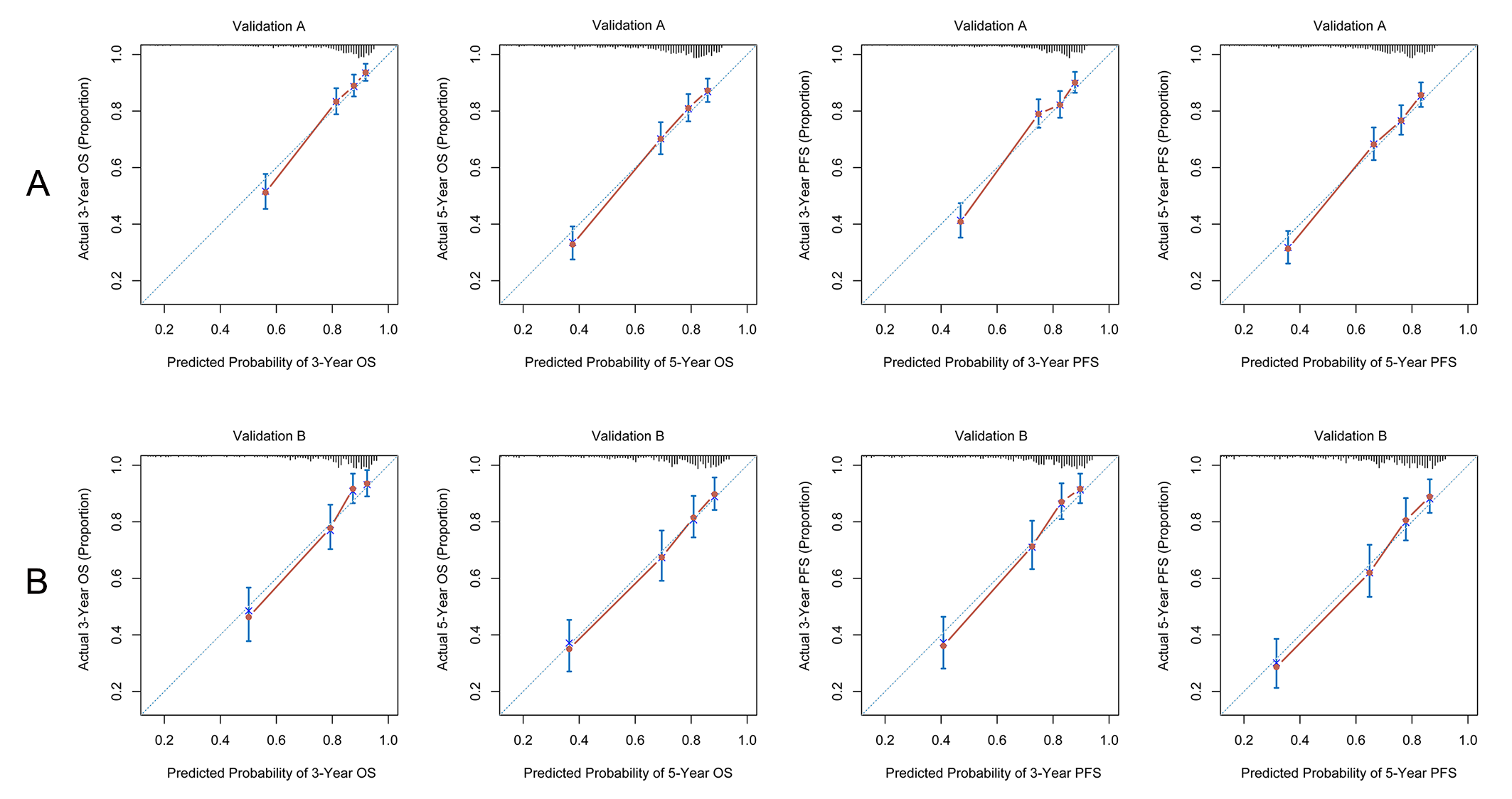
**

**Notes:** A, validation A; B, validation B.

**Table S1.** Details of postoperative complications according to modified Clavien grading system.

| Grade | Total (n=1441) | NAR | | p value |
| --- | --- | --- | --- | --- |
|  |  | Low (n = 1237) | High (n = 204) |  |
| Total complications | 299 (20.7) | 222 (17.9) | 77 (37.7) | <0.001 |
| Grade I | 147 (10.2) | 114 ( 9.2) | 33 (16.2) | 0.002 |
| Grade II | 108 ( 7.5) | 80 ( 6.5) | 28 (13.7) | <0.001 |
| Grade III | 30(2.1) | 18(1.4) | 12(5.9) | <0.001 |
| Grade IIIa | 17 ( 1.2) | 8 ( 0.6) | 9 ( 4.4) | <0.001 |
| Grade IIIb | 13 ( 0.9) | 10 ( 0.8) | 3 ( 1.5) | 0.412 |
| Grade IV | 13(0.9) | 9(0.8) | 4(2.0) | 0.084 |
| Grade IVa | 7 ( 0.5) | 7 ( 0.6) | 0 ( 0.0) | 0.603 |
| Grade IVb | 6 ( 0.4) | 2 ( 0.2) | 4 ( 2.0) | <0.001 |
| Grade V | 1 ( 0.1) | 1 ( 0.1) | 0 ( 0.0) | 0.858 |

**Table S2.** Univariate and multivariate Logistic regression analysis of complications in CRC patients.

| **Characteristic** | **Complication** | | | |
| --- | --- | --- | --- | --- |
|  | Univariate analysis | | Multivariate analysis | |
|  | HR (95%CI) | P value | HR (95%CI) | P value |
| Gender (Female) | 0.975 (0.749, 1.269) | 0.848 |  |  |
| Age (≥60 years) | 1.606 (1.240, 2.079) | <0.001 | 1.653 (1.266, 2.158) | <0.001 |
| BMI |  | 0.665 |  |  |
| Low | Ref. |  |  |  |
| Normal | 0.955 (0.648, 1.406) | 0.815 |  |  |
| High | 0.847 (0.551, 1.300) | 0.447 |  |  |
| Hypertension (Yes) | 1.407 (1.020, 1.942) | 0.037 |  |  |
| Diabetes (Yes) | 1.337 (0.818, 2.186) | 0.247 |  |  |
| NAR (High) | 2.772 (2.017, 3.810) | <0.001 | 2.298 (1.642, 3.216) | <0.001 |
| T stage (T3-4) | 1.471 (1.077, 2.009) | 0.015 | 1.335 (0.965, 1.847) | 0.081 |
| pN stage |  | 0.243 |  |  |
| N0 | Ref. |  |  |  |
| N1 | 1.267 (0.946, 1.695) | 0.112 |  |  |
| N2 | 1.197 (0.840, 1.706) | 0.320 |  |  |
| Distant metastasis (Yes) | 1.184 (0.779, 1.798) | 0.429 |  |  |
| Tumor location (Colon) | 1.219 (0.945, 1.573) | 0.128 |  |  |
| Tumor size (≥5cm) | 1.278 (0.990, 1.649) | 0.059 |  |  |
| Perineural invasion (Positive) | 1.642 (1.123, 2.400) | 0.011 | 1.528 (0.989, 2.361) | 0.056 |
| Vascular invasion (Positive) | 1.390 (1.010, 1.913) | 0.043 | 1.110 (0.768, 1.603) | 0.580 |
| Macroscopic type |  | 0.666 |  |  |
| Protrude type | Ref. |  |  |  |
| Infiltrating type | 1.134 (0.678, 1.896) | 0.632 |  |  |
| Ulcerative type | 1.143 (0.853, 1.531) | 0.372 |  |  |
| Histological grade (High/Medium) | 0.767 (0.537, 1.097) | 0.147 |  |  |
| Surgical approach (Laparoscope) | 0.572 (0.443, 0.739) | <0.001 | 0.729 (0.554, 0.959) | 0.024 |
| Operating time (median) (≥192 min) | 1.413 (1.094, 1.827) | 0.008 | 1.252 (0.953, 1.645) | 0.107 |
| Blood loss (median) (≥100 mL) | 1.619 (1.224, 2.142) | 0.001 | 1.278 (0.947, 1.726) | 0.109 |
| CEA (≥5ng/ml) | 1.289 (0.998, 1.666) | 0.052 |  |  |

**Table Note:** CRC, colorectal cancer; BMI, body mass index; NAR, neutrophil-albumin ratio.

**Table S3.** Trend test of the relationship between NAR and survival.

| PFS | | | | | | |
| --- | --- | --- | --- | --- | --- | --- |
| NAR | Model a | p value | Model b | p value | Model b | p value |
| Continuous (per SD) | 1.152 (1.085,1.223) | <0.001 | 1.1 (1.034,1.17) | 0.002 | 1.100 (1.030,1.170) | 0.006 |
| Cutoff value |  |  |  |  |  |  |
| Low | ref |  | ref |  | ref |  |
| High | 1.703 (1.396,2.077) | <0.001 | 1.384 (1.127,1.698) | 0.002 | 1.320 (1.060,1.630) | 0.013 |
| Quartiles |  |  |  |  |  |  |
| Q1 | ref |  | ref |  | ref |  |
| Q2 | 1.044 (0.823,1.325) | 0.724 | 1.038 (0.817,1.318) | 0.762 | 1.040 (0.820,1.330) | 0.740 |
| Q3 | 1.277 (1.016,1.605) | 0.036 | 1.277 (1.013,1.609) | 0.038 | 1.260 (1.000,1.600) | 0.051 |
| Q4 | 1.497 (1.197,1.871) | <0.001 | 1.354 (1.08,1.697) | 0.009 | 1.330 (1.050,1.690) | 0.019 |
| p for trend |  | <0.001 |  | 0.002 |  | 0.007 |
| OS | | | | | | |
| NAR | Model a | p value | Model b | p value | Model b | p value |
| Continuous (per SD) | 1.162 (1.094,1.235) | <0.001 | 1.112 (1.043,1.184) | 0.001 | 1.090 (1.010,1.170) | 0.018 |
| Cutoff value |  |  |  |  |  |  |
| Low | ref |  | ref |  | ref |  |
| High | 1.769 (1.444,2.167) | <0.001 | 1.438 (1.167,1.771) | 0.001 | 1.300 (1.040,1.620) | 0.019 |
| Quartiles |  |  |  |  |  |  |
| Q1 | ref |  | ref |  | ref |  |
| Q2 | 1.106 (0.863,1.418) | 0.426 | 1.08 (0.842,1.386) | 0.545 | 1.060 (0.830,1.370) | 0.626 |
| Q3 | 1.328 (1.045,1.687) | 0.02 | 1.319 (1.035,1.68) | 0.025 | 1.260 (0.980,1.610) | 0.069 |
| Q4 | 1.603 (1.269,2.023) | <0.001 | 1.443 (1.14,1.826) | 0.002 | 1.320 (1.020,1.690) | 0.032 |
| p for trend |  | <0.001 |  | 0.001 |  | 0.015 |

**Notes:**

Model a: No adjusted.

Model b: Adjusted for gender, age, and BMI.

Model c: Adjusted for gender, age, BMI, hypertension, diabetes, T stage, N stage, metastasis, tumor location, tumor size, perineural invasion, vascular invasion, macroscopic type, differentiation, surgical approach, operating time, blood loss.

**Table S4.** The clinicopathological Features of two validation cohorts in CRC patients.

| Features | Validation a  (n = 1009) | Validation b  (n = 432) | P value |
| --- | --- | --- | --- |
| Gender(male) | 641 (63.5) | 263 (60.9) | 0.372 |
| Age (≥60) | 506 (50.1) | 218 (50.5) | 0.959 |
| Age (mean (SD)) | 58.02 (13.24) | 58.35 (12.95) | 0.66 |
| BMI (median [IQR]) | 22.07 (19.96, 24.44) | 21.78 (19.92, 24.22) | 0.258 |
| BMI |  |  | 0.632 |
|  | 122 (12.1) | 60 (13.9) |  |
|  | 603 (59.8) | 251 (58.1) |  |
|  | 284 (28.1) | 121 (28.0) |  |
| Hypertension (Yes) | 172 (17.0) | 69 (16.0) | 0.672 |
| Diabetes (Yes) | 61 ( 6.0) | 29 ( 6.7) | 0.718 |
| T stage |  |  | 0.752 |
|  | 34 ( 3.4) | 16 ( 3.7) |  |
|  | 230 (22.8) | 88 (20.4) |  |
|  | 537 (53.2) | 233 (53.9) |  |
|  | 208 (20.6) | 95 (22.0) |  |
| N stage |  |  | 0.575 |
|  | 565 (56.0) | 243 (56.2) |  |
|  | 285 (28.2) | 113 (26.2) |  |
|  | 159 (15.8) | 76 (17.6) |  |
| Clinical distant metastasis (Yes) | 89 ( 8.8) | 48 (11.1) | 0.208 |
| TNM stage |  |  | 0.221 |
|  | 206 (20.4) | 78 (18.1) |  |
|  | 326 (32.3) | 154 (35.6) |  |
|  | 388 (38.5) | 152 (35.2) |  |
|  | 89 ( 8.8) | 48 (11.1) |  |
| Perineural invasion (Yes) | 109 (10.8) | 40 ( 9.3) | 0.431 |
| Vascular invasion (Yes) | 188 (18.6) | 59 (13.7) | 0.026 |
| Macroscopic type |  |  | 0.393 |
| Protrude type | 276 (27.4) | 130 (30.1) |  |
| Infiltrating type | 84 ( 8.3) | 29 ( 6.7) |  |
| Ulcerative type | 649 (64.3) | 273 (63.2) |  |
| Differentiation (Poor) | 142 (14.1) | 48 (11.1) | 0.15 |
| Tumor location (Rectal) | 514 (50.9) | 222 (51.4) | 0.922 |
| Tumor size (median [IQR]) | 4.50 (3.50, 6.00) | 4.50 (3.50, 6.00) | 0.049 |
| CEA (High) | 412 (40.8) | 182 (42.1) | 0.689 |
| Surgical method (Endoscopic) | 592 (58.7) | 245 (56.7) | 0.527 |
| Operation time (median [IQR]) | 190.00(149.00, 246.00) | 184.00(150.00, 240.00) | 0.837 |
| Intraoperatve blood soss(median [IQR]) | 100.00 (50.00, 200.00) | 100.00 (50.00, 200.00) | 0.566 |
| Length of stay (median [IQR]) | 17.00 (11.00, 21.00) | 17.00 (11.00, 21.00) | 0.891 |
| Radiotherapy (Yes) | 97 ( 9.6) | 37 ( 8.6) | 0.597 |
| Chemotherapy (Yes) | 467 (46.3) | 190 (44.0) | 0.456 |

**Table Note:** CRC, colorectal cancer; BMI, body mass index; NAR, neutrophil-albumin ratio.
